# Understanding the Relationship Between Biotherapeutic Protein Stability and Solid–Liquid Interfacial Shear in Constant Region Mutants of IgG1 and IgG4

**DOI:** 10.1002/jps.23822

**Published:** 2013-12-19

**Authors:** Roumteen Tavakoli-Keshe, Jonathan J Phillips, Richard Turner, Daniel G Bracewell

**Affiliations:** 1 The Advanced Centre for Biochemical Engineering, University College London, Torrington PlaceLondon, WC1E 7JE, UK; 2 MedImmune, Granta ParkCambridge, CB21 6GH, UK

**Keywords:** protein aggregation, protein structure, molecular modeling, IgG antibody, thermal analysis, biopharmaceuticals characterization, calorimetry (DSC), *in silico* modeling

## Abstract

Relative stability of therapeutic antibody candidates is currently evaluated primarily through their response to thermal degradation, yet this technique is not always predictive of stability in manufacture, shipping, and storage. A rotating disk shear device is proposed that produces defined shear conditions at a known solid–liquid interface to measure stability in this environment. Five variants of IgG1 and IgG4 antibodies were created using combinations of two discrete triple amino acid sequence mutations denoted TM and YTE. Antibodies were ranked for stability based on shear device output (protein decay coefficient, PDC), and compared with accelerated thermal stability data and the melting temperature of the CH2 domain (*T*_m_1) from differential scanning calorimetry to investigate technique complimentarity. Results suggest that the techniques are orthogonal, with thermal methods based on intramolecular interaction and shear device stability based on localized unfolding revealing less stable regions that drive aggregation. Molecular modeling shows the modifications’ effects on the antibody structures and indicates a possible role for Fc conformation and Fab-Fc docking in determining suspended protein stability. The data introduce the PDC value as an orthogonal stability indicator, complementary to traditional thermal methods, allowing lead antibody selection based on a more full understanding of process stability. © 2013 The Authors. *Journal of Pharmaceutical Sciences* published by Wiley Periodicals, Inc. and the American Pharmacists Association J Pharm Sci 103:437–444, 2014

## INTRODUCTION

Minimizing aggregation in protein therapeutics is a major challenge in drug development, involving a thorough understanding of the factors influencing protein stability over the product lifetime.^1^,^2^ Aggregation is a critical regulatory concern when choosing a lead candidate for production because of the health risks associated with injecting aggregated proteins into patients,^3^,^4^ and their ability to neutralize antibodies and inhibit the efficacy of the product.^5^ Current guidelines advise measuring aggregation using the light obscuration method described in the European Pharmacopoeia methods 2.9.19^6^ and the US Pharmacopoeia (USP) <788>.^7^ These state that particulates greater than 10 μm in size should be controlled at or below 6000 particles per container, whereas particulates greater than 25 μm should be controlled at less than 600 particles per container.^8^

One common approach to mitigate particulate formation and other degradation phenomena is to determine relative stability with the aid of temperature hold studies, often after screening many candidates using differential scanning calorimetry (DSC).^9^ Other methods for looking at the stability of candidates, which rely on structural characterization are available such as circular dichroism^10^ and spectroscopy techniques.^11^,^12^ These methods give more specific information about the candidates but cannot easily be used to rank antibody stabilities. Alternative methods are emerging that measure protein stability based on degradation by interfacial effects. These methods allow assessment of the impact of solid–liquid^13^ or air–liquid^14^ interfaces at the same time as ranking protein stability in formulation conditions. At present, however, there is little standardization of such methods and no consensus on which is the best to use.

Significant amounts of aggregates can form around nuclei over timeframes relevant to modern pharmaceuticals and under the stress conditions they are exposed to.^15^,^16^ Stress is commonly used as a generic term unless specifically stated to describe a range of forces (e.g., hydrodynamic, chemical, thermal, and interfacial) exerted on a protein in different environments.^16^–^18^ For example, one study showed that the aggregate produced was dependent upon whether the solution environment was shaken or stirred.^15^,^19^ These external stresses can directly disrupt the native conformation of the protein molecule, causing changes to the surface charge and hydrophobicity of the molecule,^20^ allowing binding to other proteins.^21^ In general, the maximum shear rate created during downstream processing is around 20,000 s^−1^.^22^ Such mechanical stress is generated by pumping, filtration, mixing, fill-finishing, shipping or shaking,^23^ affecting protein stability,^24^ and resulting in a loss of soluble protein.^25^

The effect of shear stress on the aggregation rate of proteins has produced conflicting reports seemingly because of the presence or absence of different interfaces. There are some suggestions that shear causes minor conformational changes to the native structure of the monomer.^26^ Monomeric mAb showed no aggregation after 30–51 ms exposure to shear rates up to 250,000 s^−1^, with only reversible aggregates in the 40–60 unit range observed after 300 s at 20,000 s^−1^ shear. A second sample with an aggregate population of 17% was not significantly altered by the shear rate.^22^ Although shear alone appears not to cause formation of large aggregates during mixing and adsorption to stainless steel surfaces shows only small levels of aggregation,^27^ together the effects of these forces can more be pronounced. Previous work highlights how at solid–liquid interfaces both the physiochemical properties of the solid interface and the shear above that boundary directly impact the rate of antibody aggregate formation.^28^ This infers the hydrodynamic environment directly above the surface is critical to rate of aggregate formation. The rotating disk surface adsorption shear device used in this work was developed at UCL to mimic loss of protein to aggregation experienced through solid–liquid interfacial effects particularly during fill-finish operations.^13^ Monomer loss from prolonged operation of the device follows first-order exponential decay and is protein specific, dependent on both disk speed and surface material.^28^ By having a well-controlled shear and interface environment and using the coefficient of decay of nine sample points over 2 h of shear device operation, reproducible results can be achieved using this method.

The most prominent class of biopharmaceuticals are IgG antibodies, which are globular plasma glycoproteins with a mass of approximately 146 kDa^29^ consisting of two light and two heavy chains, folded to form domains of two beta sheets that create a sandwich shape held together by conserved disulfide bonds and other noncovalent interactions. The two identical γ heavy chains contain around 450 amino acids and consist of three constant and one variable domain. The light chains contain around 215 amino acids and again consist of a constant and a variable domain. These variable domains are the origin of protein specificity.^30^ The IgG4 subtype structure differs from the IgG1 subtype in that they are unable to cross-link two antigens because of the monovalent behavior.^31^ It has been suggested this behavior is because of an amino acid change from proline to serine in the core of the hinge of IgG1^32^ resulting in less disulfide bond formation. The molecule dissociates from the hinge and produces identical monomeric halves, each containing a light and heavy chain. Bispecificity is observed when two different IgG4 monomeric halves reform to give a whole biclonal antibody. This prevents precipitation of the purified antibodies^33^ under normal conditions because they are unable to cross-link two antigens.

Considerable work has gone into defining the role of the hinge on the effector functions of an antibody. For IgG, this hinge can generally be divided into upper, middle, and lower regions. The middle hinge stretches from the first to the last cysteine that form disulfide bridges and is believed to be rigid because of interheavy chain disulfide bridging and the subsequent formation of polyproline helices.^34^ It is considered that the hinge region acts as a spacer, effecting segmental flexibility and allowing the Fab arms to move in relation to the Fc.^35^ A study showed that changes in the amino acid sequences could affect the rigidity of the hinge, which in turn affected the effector function of the antibody.^36^ Further studies show that IgG4 has less flexibility in the hinge region than IgG1,^37^ which correlates with a reduction in stability between the two subgroups.^37^,^38^ This also correlates with a greater hydrodynamic radius of the IgG4 subtype.

**Figure 1:**
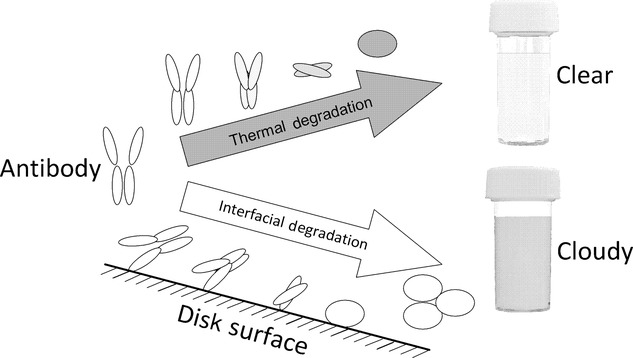
Diagram representing comparability of thermal degradation and interfacial shear-induced aggregation for determining relative stability. The thermal degradation route is based on work on nonnative protein aggregation by Roberts and co-workers,^41^,^42^ whereas the interfacial route builds upon this background with work by Biddlecombe et al.^28^

In this study, the effects of controlled antibody modifications TM^39^ and YTE^40^ on a drug candidate's secondary and tertiary structures and overall charge were evaluated. By using the solid–liquid interfacial shear device, the relative stability of protein candidates was measured. This technique was compared with melting temperature (*T*_m_) data from DSC and the results from a 4-week accelerated stability study at 40°C to give relative stability based on thermal parameters. This comparison could be used to determine whether the shear device method could be used as an orthogonal method for lead antibody screening (Fig. 1).

Molecular modeling techniques^43^ were then employed to help determine the effect of the modifications on the proteins’ secondary and tertiary structures, to help explain any trends seen in the relative stabilities of the antibodies using surface charge and spatial aggregation propensity (SAP) information.

## MATERIALS AND METHODS

### Antibodies

Five sets of human monoclonal antibodies, three IgG1 and two different IgG4, were expressed by CHO cells and purified using protein A and cation chromatography at MedImmune (Cambridge, UK). Candidates were formulated in a stability buffer containing l-histidine and d(+)-trehalose pH 5.5 at concentrations of 1 mg/mL, and with an overall purity of at least 99%. Antibodies were labeled as IgG1—WT, YTE, and TM YTE—and IgG4—WT and YTE.

### Size-Exclusion High-Pressure Liquid Chromatography

A TSKgel3000SWXL (TOSOH Biosciences, Tokyo, Japan) size-exclusion column (7.8 × 300 mm^2^) was run using an Agilent 1100 HPLC system (California) comprising degasser, solvent pump, injector, temperature controller, and a fixed-wavelength (DAD) UV detector. The mobile phase was 200 mM sodium phosphate buffer pH 7.4 used at a flow rate of 1 mL/min. Samples were centrifuged at 18,000 g for 10 min prior to size-exclusion high-pressure liquid chromatography (SE-HPLC). Injection volume for samples was set at 20 μL and the eluate from the column was monitored at 280 nm. Peaks were subsequently integrated and antibody monomer concentration was calculated as a proportion of the peak area prior to exposure to the stress of the shear device, taking into account dilution effects from the displacement-based sampling method of the custom made shear device.

### Shear Device

The shear device generated a defined high-shear environment used to simulate the effect of surface adsorption and shear forces encountered during processing. The round device chamber was constructed of stainless steel (30 × 17 mm^2^) containing a stainless steel disk (26 × 8 mm^2^). It applied high-shear rates while eliminating the air–liquid interface and controlling temperature.^13^

### Shear Device Operation

The shear device was filled with 7 mL of antibody solution from a syringe through a one-way valve on the top face and all air was excluded. The temperature was controlled at 17°C by circulation of water at predetermined temperatures with a fixed flow rate controlled by a peristaltic pump (Beckman Coulter, Brae, California) feeding into the cooling jacket. The antibodies were sheared using a 240-grit stainless steel disk at 6000, 7500, 9000, and 12,000 rpm equivalent to a maximum shear strain rate of 1.59, 1.98, 2.38, and 3.17 × 10^4^ s^−1^, respectively, for 2 h. Samples (100 μL) were taken every 15 min by displacing the sample chamber contents using the l-histidine stability buffer. Each sample resulted in a 1.4% dilution of the chamber solution with a final dilution over 2 h of around 10%. This dilution factor was subsequently accounted for in calculations.

### Differential Scanning Calorimetry

Differential scanning calorimetry studies were performed using a Microcal VP-Capillary DSC (GE Healthcare, Bucks, UK). Samples were prepared in the stability buffer at concentrations of 1 mg/mL. The change in the heat capacity of the sample was measured in comparison with a sample of buffer alone as samples were heated from 45°C to 95°C at a rate of 60°C/h. The heat capacity data were normalized and corrected for the baseline.

### Accelerated Stability Studies

Antibodies were held in stability buffer at 10 mg/mL at a temperature of 40°C. Samples were analyzed at 0, 2, and 4 weeks from the start of the experiment using HPLC and the monomer percentage determined.

### Molecular Modeling

Modeling and analysis of the IgG Fc was performed using PyMol (Schrodinger, Munich, Germany) and Discovery Studios (Accelyrys, California) software.

## RESULTS AND DISCUSSION

### Shear Stability of mAb Candidates: A Divide Between IgG1 and IgG4

**Figure 2:**
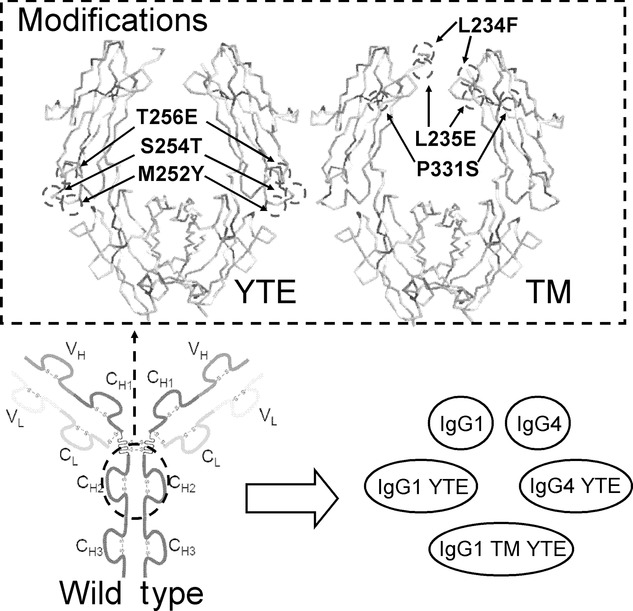
Diagram showing the site of each mutation on the structure of the antibody and the resultant antibody formats used for comparison. 3D protein structure taken from PDB, filename 2DQT.

**Figure 3:**
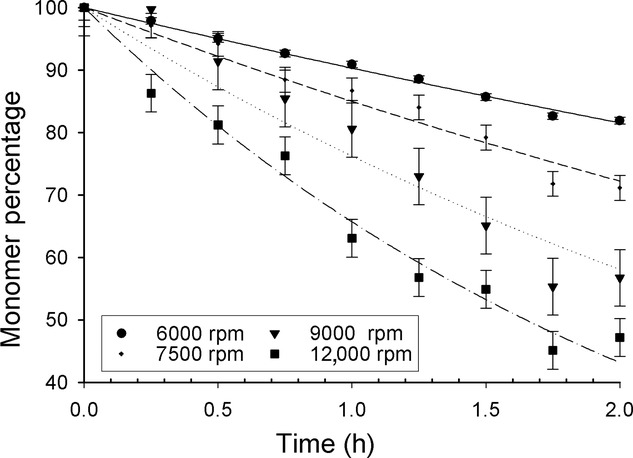
The effect of disk speed on perceived monomer loss in shear device. Percentage of original monomer concentration remaining over time of shear for 1 mL/mg sample of IgG1 TM YTE in l-histidine and d(+)-trehalose buffer in shear device at 6000, 7500, 9000, and 12 000 rpm. Error bars show the standard error of the sets for their fit with the regression line.

The candidates for stability studies were produced from the IgG1 and IgG4 formats of an antibody. These formats were modified using combinations of two discrete, defined triple amino acid sequence mutations, TM^39^ and YTE,^40^ to give five variants with known pharmacological properties (Fig. 2). The five different antibodies were processed in the shear device at 6000, 7500, 9000, and 12,000 rpm. The antibody concentration selected was 1 mg/mL based on previous work and experimental evidence showing little change in the protein decay coefficient (PDC) between 0.5 and 10 mg/mL and a desire to minimize sample consumption. Each processed sample was cloudy when removed from the chamber, indicating the presence of aggregate. The insoluble material was removed by centrifugation prior to SE-HPLC. The area under the monomer curve at 15 min intervals was fitted to an exponential decay curve:
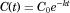
where *C*(*t*) is the monomer concentration (mg/mL) at time *t* (h), *C*_0_ is the monomer concentration at time zero and *k* is the PDC (h^−1^). Statistical *t*-tests performed on the groups of results from each shear rate gave a confidence level of 97.5% that the trend seen was significant. The monomer reduction profiles for IgG1 TM YTE (Fig. 3) are shown as an example of the profiles seen. Greater monomer loss occurred at increased disk speeds indicating that the stability of the antibody was affected by processing at a range of disk speeds in the device.

The PDC values for different disk speeds were used to compare monomer losses between each run. These were fitted to a second-order regression to highlight clear trends that showed the antibodies to have varying levels of sensitivity to increases in the disk speed (Table1), caused by their different inherent stabilities in these conditions. Overall, the IgG1 subtype candidates had a lower monomer loss than the IgG4s at any given disk speed. There was however more variability between mutants in the IgG1 PDC values at each disk speed, with the IgG4 candidates showing similar, high PDC values, possibly indicating their interfacial shear stability is already compromised. This is likely related to the lack of a stable hinge region holding the antibody halves together.^32^ Two distinct lots of the IgG4 YTE were separately produced and purified to test the reproducibility of the interfacial shear device result. The results showed that the error of the PDC-derived decreases with disk speed, creating a need to compromise between ensuring reproducibility of data without losing sensitivity of the technique. A disk speed of 9000 rpm was chosen to be the optimum level.

**Table 1:** Protein Decay Coefficient Values at Different Disk Speeds for All Candidates

	Protein Decay Coefficient (h^−1^)			
Antibody	6000	7500	9000	12,000
IgG1 WT	0.095	0.136	0.179	0.285
IgG1 YTE	0.077	0.108	0.115	0.223
IgG1 TM YTE	0.102	0.163	0.272	0.420
IgG4 WT	0.147	0.209	0.285	0.476
IgG4 YTE 1	0.168	0.212	0.298	0.471
IgG4 YTE 2	0.184	0.219	0.305	0.470

PDC is the coefficient of exponential decay for monomer loss curve of antibodies prepared at 1 mg/mL in l-histidine and d(+)-trehalose buffer after 2 h of shear device operation. IgG4 YTE duplicates shown for the measure of reproducibility of technique.

The increase in soluble monomer loss with disk speed could be because of an increase in its loss to aggregate nuclei in solution or to an increase in the rate of the dissociation of aggregate precursors from the disk surface, resulting in an increased rate of aggregation. The work also supported previous observations that an increase in large aggregates (based on the increased opalescence of the solution) did not serve to increase the rate of monomer loss.^13^ The relatively steady rate of monomer loss differs from some reported routes of protein aggregation where the level of monomer loss seems to be low for a nucleation phase, followed by rapid fibrillation,^44^,^45^ indicating the presence of different mechanisms of aggregation.

### DSC of mAb Candidates: The IgG1 Subtype Displays Greater Thermo-Stability than the IgG4 Subtype

**Figure 4:**
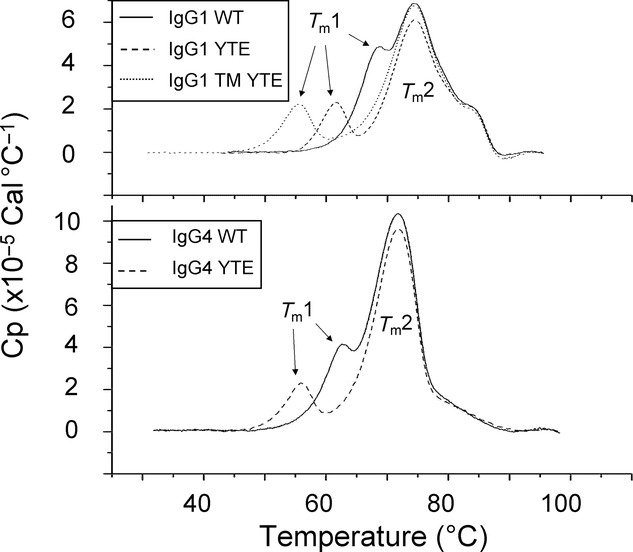
The effect of modifications on thermal stability determined by DSC. DSC profiles to show heat capacity of 1 mg/mL samples of IgG1 WT, YTE, TM YTE and IgG4 WT and YTE in l-histidine and d(+)-trehalose buffer (pH 5.5).

The DSC profiles (Fig. 4) showed the antibodies have two clearly resolved transitions, each peak in the trace giving a *T*_m_ value for the unfolding of each protein domain. The first peak (*T*_m_1) representing the unfolding transition of the CH2 domain was well resolved for most of the antibody candidates. The Fab and CH3 were unresolved in the second unfolding transition (*T*_m_2) with a higher enthalpy than in *T*_m_1, possibly indicating stronger interactions between subdomains in this region.^46^ As expected, the modifications in the CH2 change the *T*_m_1 value significantly.

The *T*_m_1 values for the antibody variants showed a greater range than the *T*_m_2 values giving different resolutions of the peaks. Some previous work intimates that peak resolution is related to the level of interdomain interaction in the antibody,^47^,^48^ suggesting that the closer and less resolved the two peaks appear, the more interaction they have with each other during unfolding. Other sources however dispute this and state that the individual domain peaks unfold independently^49^ and just happen to overlap because of similar unfolding temperatures. IgG1 WT displayed the least resolution of the subtype, with closely associated peaks with a high *T*_m_1 value. IgG1 YTE and TM YTE had similar profiles with two distinct individual peaks, but IgG1 TM YTE had a lower *T*_m_1 value. It is interesting to note that IgG4 WT also had a near identical *T*_m_2 value to IgG4 YTE. The *T*_m_1 value for IgG4 WT was much higher than for IgG4 YTE, giving less resolution between the peaks. The emerging shoulder on the right of *T*_m_2 unfolding transition for the IgG1 subtype compared with the trailing edge on the corresponding transition for the IgG4 subtype showed greater stability in the CH3 region of the IgG1. It is speculated that greater areas of the peaks could be a result of IgG4's ability to rotate and translate more than the IgG1 because of the IgG4's aforementioned weaker linker hinge in the Fc.

The *T*_m_1 value is predominantly used in the industry as an approximation to screen the relative stability of antibodies and to determine the optimal temperature to perform accelerated stability testing,^50^ although the use of this over *T*_m_2 is being increasingly challenged. The comparison of *T*_m_1 values with the PDC comparator obtained from the interfacial shear device monomer loss data would show how related the two stability readings are. On the basis of the greater enthalpy, the *T*_m_2 value should represent the major unfolding transition of the molecules. It has also been reported that although changes to the protein structure at *T*_m_1 values are reversible, changes that occur at *T*_m_2 values are irreversible.^41^ This could mean that the *T*_m_2 value is in fact more pertinent to predicting protein aggregation; however, the data showed negligible difference with *T*_m_2 readings for the modifications, although the IgG1 and IgG4 populations did fall into two distinct subsets. It is logical that the modifications located in the CH2 region would not greatly affect the stability of the Fab transition. The weak linker region in IgG4 molecules could account for the lower stability seen in general for the IgG4 candidates. This region has reduced interheavy chain disulfide bridge stability, allowing the molecule to detach into two monomeric halves.^32^ The resistance of the whole molecule to degradation must be greater than the sum of the two halves. It should be stated that the stability of these molecules is not necessarily related to their efficacy.

### Accelerated Thermal Stability of mAb Candidates: IgG4 Candidates Show Reduced Thermal Stability

**Figure 5:**
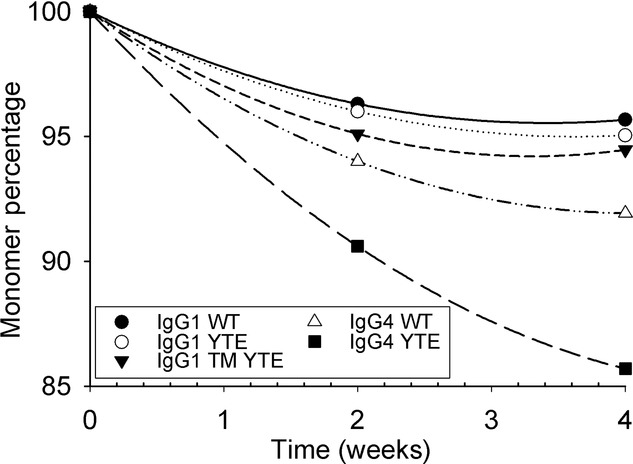
The effect of modifications on accelerated stability studies. Monomer percentage determined by SE-HPLC at 0, 2, and 4 weeks using 10 mg/mL antibody samples in l-histidine and trehalose buffer held at 40°C.

The accelerated stability results did differentiate between the IgG1 and IgG4 candidates (Fig. 5). In line with the previous reports,^51^ there were minimal amounts of fragmentation present in the thermally denatured samples, with the vast majority of monomer loss through aggregation mechanisms. Curves for loss were fitted to a second-order polynomial curve, showing a clear trend for each antibody. IgG4 candidates showed reduced thermal stability in comparison with IgG1 candidates. Because the trends reported did not converge, the value of the monomer percentage at week 4 was used for further analysis of the data.

### Comparison of Shear Device and Thermal Stability: Interfacial Shear Device Promotes Formation of High-Molecular-Weight Aggregates

**Figure 6:**
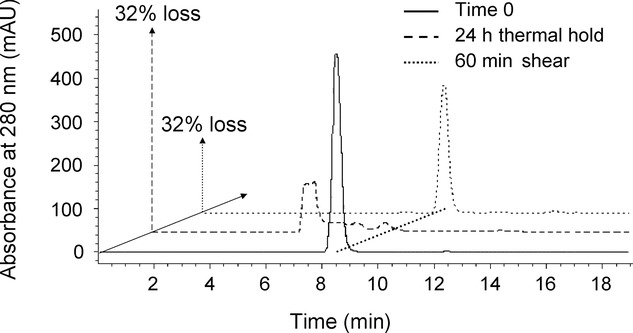
Comparison of HPLC traces for 5 mg/mL IgG1 WT samples processed at different conditions. (a) ____ Before processing, (b) —– after 24 h hold at pH 4 and 40°C, (c) after 60 min shear at 9000 rpm.

HPLC traces from samples aggregated through the interfacial shear device were compared with those that had been degraded through thermal routes (Fig. 6). Interfacial shear-device-degraded samples showed a reduction of monomer and no measurable increase in fragments or multimers. This indicates that the antibody had aggregated and formed much larger particles that had begun to fall out of solution, as shown by the increased opaqueness of samples taken from the device. This implies that the multimers are either very short lived, making them difficult to detect, or they are formed at the solid–liquid interface prior to release into the bulk solution. Thermally denatured samples showed a reduction of monomer, with the majority of the antibody found as low-molecular-weight multimer, suggesting a more classically observed nucleation mechanism for the aggregation.^15^

### The Relationship Between PDC, DSC, and Accelerated Stability: Values Look at Different Aspects of Stability

**Figure 7:**
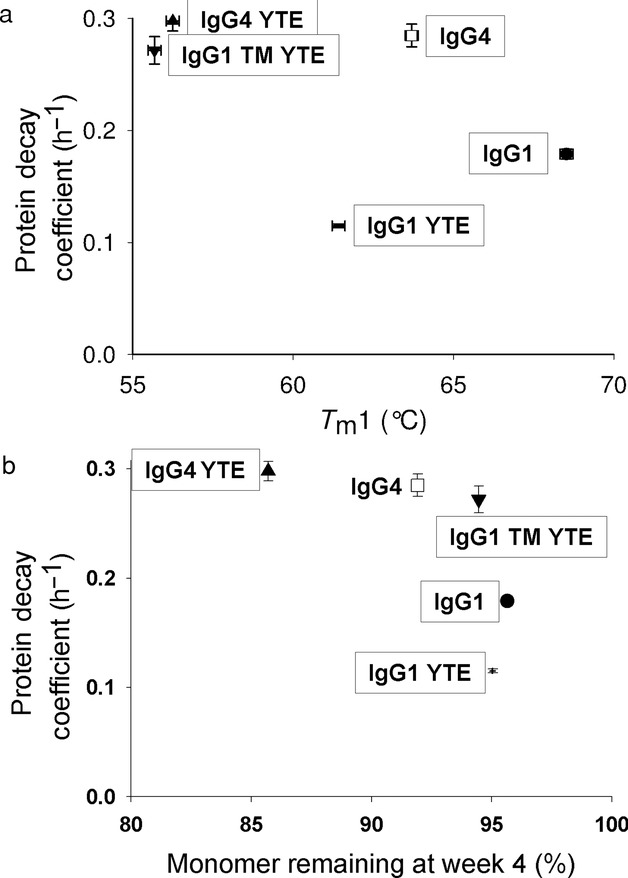
(a) Comparison of PDC with *T*_m_1 for all candidates at a disk rate of 9000 rpm. *y*-Axis error bars are for standard error of PDC fit, and *x*-axis error bars are for internal error of DSC. (b) Comparison of monomer remaining at week 4 in accelerated stability results, determined by SE-HPLC and the PDC as determined with custom shear device method for all candidates at a disk rate of 9000 rpm. *x*-Axis error bars are for standard error of PDC fit.

The correlation of the thermal stability measured by DSC with conformational stability measured by adsorption to solid surfaces in a high-shear environment was assessed. Different antibodies are shown (Fig. 7a) in terms of their first melting temperature, *T*_m_1, which represents unfolding of the CH2 region. This comparator is predominantly used in industrial screening of antibody candidates to determine the most stable candidates for accelerated stability studies. Comparison of the two stability indicators showed that there was no correlation between the thermal and the surface adsorption-based stability. The IgG4 candidates did show that although they were the least stable in the shear device, they had relatively good thermal stability for this transition. The lack of correlation suggests that different mechanisms of degradation are involved in the two testing methodologies.

Comparison of PDC using 1 mg/mL protein with monomer percentage determined from accelerated stability studies at 40°C (Fig. 7b) using 10 mg/mL protein revealed little trend between the two stability indices. The concentrations of antibody solution were kept at the standard operating conditions of each method. The IgG4 accelerated stability data supported the *T*_m_2 results from the DSC analysis, highlighting the inability of the *T*_m_1 values to differentiate between IgG1 and IgG4. IgG1 YTE was shown to be significantly more stable to damage via interfacial effects than IgG1 WT, yet slightly less thermally stable. This indicates that the two methods are not directly comparable.

A comparison of the *T*_m_1 value and the accelerated stability method also shows no correlation. Similar to the comparison of the shear device with *T*_m_1, the *T*_m_1 shows IgG4 candidates to be comparatively stable, whereas the accelerated stability method indicates them to be significantly less stable than the IgG1. Looking across these comparisons, the three methods rank stability differently based on distinct mechanisms of loss of structural integrity.

### Insights Based on Molecular Modeling

**Figure 8:**
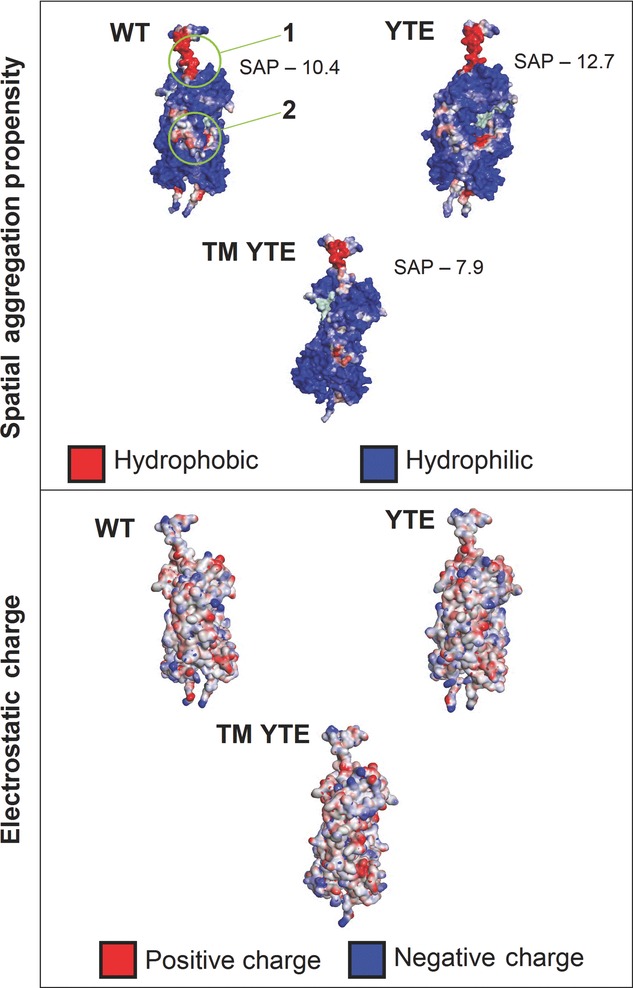
Comparison of SAP and surface charge of IgG1 WT Fc with the YTE and TM YTE modified IgG1 Fc's. Site 1 is the site of the *T*_m_ modification, and site 2 is the site of the YTE modification. Numerical value of the SAP of site 1 is shown.

Molecular models of the IgG1 Fc and hinge region, with two internal fucosylations, were analyzed for their SAP and electrostatic surface charge displayed in Figure 8. There are known docking postures of the Fab onto the hinge and the superior surface of the Fc. The CH1-1 loop of the Fab is more intrinsically disordered than even vhCDR3^52^ and is oriented toward the central hinge in the full-length mAb crystal structure,^53^ though it does not have electron density in the model 1HZH (PDB). This, together with high sequence variability in this loop, would imply that it has no conserved interactions with the hinge region. The minor hinge, however, has greater sequence conservation and is shown in a stably docked posture in the full-length antibody crystal structure,^53^ orientated with the minor hinge of the light chain forming an interface with the location of the first two mutants of the “TM”.

The model results of the three modifications show there is no significant difference shown in the electrostatic results, with the overall surface charges of the proteins remaining the same. This shows that the difference in stability is not from electrostatic interactions, a key component of colloidal stability. The SAP models do however show a very noticeable difference in the hydrophobicity of the hinge region of the Fc with the rest of the molecule not showing any significant differences. The increase in the hinge SAP scores shown in Figure 8 correlates with a reduction in the PDC (improved stability), suggesting this is the area of the molecule responsible for the change in stability documented in this instance. It is postulated that having a more hydrophilic upper hinge would reduce the range of motion of the Fab in respect to the Fc, giving a more rigid antibody structure that is less able to fold in on itself to conserve hydrophobic sections on the Fc. In the case of the YTE, this allows the enhanced binding to the Neonatal Fc Receptor, but also seems to allow easier degradation through adsorption at solid–liquid interfaces. It is suggested that the mobility of the hinge region is very important to not only in exposing effector functions of the Fc,^36^ but also in keeping the antibody stable in solution.

## CONCLUSIONS

The systematic use of known monoclonal antibody variants of IgG1 and IgG4 subtypes reveals the complexity of protein stability in solution. The three testing methods used, DSC, accelerated stability at 40°C, and stability to solid–liquid interfaces, all provided different rankings of stability, inferring differing mechanisms of damage predominant in each. The shear device provides an orthogonal approach that can be used as part of an overall screening process for protein stability. This provides the basis to characterize the critical routes of degradation encountered during therapeutic protein processing and storage. The shear device gives relative stability for routes involving shear and solid–liquid interfaces, which are present in many different steps of protein purification and in transport and storage of drug product.

The correlation of the molecular modeling results with the shear device results not only helps to explain the reason behind the change in stabilities, but underlines the benefits of using *in silico* methods for the evaluation of drug candidates in the future.^54^

A broader knowledge of antibody characteristics earlier in the drug lifecycle would allow candidates to be selected for stability through the primary recovery and downstream processing stages of production and will allow better analysis of long-term storage stability. Increased stability is however not an indicator for increased product efficacy, another factor that should be considered.
